# X chromosome inactivation, X-linked disorders, and cancer

**DOI:** 10.3389/fgene.2026.1820203

**Published:** 2026-04-30

**Authors:** Consuelo Salas-Labadía, Patricia Pérez-Vera, Fernando Gómez-Chávez, Ximena Pérez Baena, Daniel Martínez Anaya, Jorge Colín Rubio, María del Pilar Navarrete-Meneses

**Affiliations:** 1 Laboratory of Genetics and Cancer, National Institute of Pediatrics, Mexico City, Mexico; 2 Laboratorio de Enfermedades Osteoarticulares e Inmunológicas, Sección de Estudios de Posgrado e Investigación, Escuela Nacional de Medicina y Homeopatía del Instituto Politécnico Nacional, Mexico City, Mexico; 3 Department of Human Genetics, National Institute of Pediatrics, Mexico City, Mexico

**Keywords:** cancer, skewed inactivation, X-chromosome, X-chromosome inactivation, X-linked disease

## Abstract

X chromosome inactivation is an essential process that compensates for gene dosage differences between men and women. During early embryogenesis, one of the two X chromosomes in females is randomly selected for transcriptional silencing, inactivating either the maternal or paternal chromosome. This process makes the functional genetic information in females equivalent to a single X chromosome, as in males. Usually, X inactivation occurs in approximately 50% of maternal and 50% of paternal X chromosomes. However, deviations from this ratio can occur, resulting in skewed X inactivation. In women carrying pathogenic variants on the X chromosome—thus presenting X-linked syndromes—such skewing can lead to a wide range of phenotypic manifestations, making X inactivation an important subject of study. Moreover, several X-linked syndromes have been associated with an increased risk of various types of cancer. This risk is influenced not only by specific pathogenic variants but also by mechanisms such as defective X inactivation, which has itself been linked to tumor development. This review compiles both historical and recent findings on X inactivation and its relationship with cancer. It provides an updated overview of the X chromosome inactivation mechanism, a summary of X-linked disorders associated with cancer risk, a discussion of X chromosome involvement in tumorigenesis, an examination of cancer-related genes on the X chromosome, and information on sexual dimorphism in cancer.

## 1 X chromosome inactivation

Human females and males normally carry 22 pairs of autosomal chromosomes (1–22), which are the same in both sexes, and one pair of sex chromosomes (X and Y), which differ between the sexes. Females carry two copies of the X chromosome, and males carry one copy of the X chromosome and one copy of the Y chromosome. The X chromosome represents approximately 5% of the genome and contains approximately 1,500 genes (Achilla et al., 2022). Few genes on the X chromosome show homology with genes on the Y chromosome. The X chromosome dosage difference between the sexes is overcome by inactivating one X chromosome in every cell of females. In this way, XX females equalize the dosage of X-linked genes with that in XY males (Achilla et al., 2022; Lyon, 1961; Spatz et al., 2004).

X chromosome inactivation (XCI) is thus an important process in which one of the two X chromosomes, either maternal or paternal, is silenced. This was first proposed in 1961 by Mary Lyon, who hypothesized that equalization of X-linked gene dosage between males and females is achieved by the transcriptional silencing of one X chromosome (Lyon, 1961). XCI initiates with a process of counting the number of X chromosomes, ensuring that all but one X chromosome becomes silent. Then, randomly, the X chromosome to be inactivated is chosen, assuring that the X chromosome inherited from each parent is silenced in approximately 50% of the cells. Finally, the selected X chromosome is memorized, so that the cells of the subsequent round of divisions inherit the same inactivation (Spatz et al., 2004).

X chromosome silencing involves steps of epigenetic modification. This process is initiated in the X-inactivation center (XIC), a specific site on the long arm of the X chromosome (Xq13) that contains the X-inactive specific transcript (*XIST*) gene. The *XIST* gene encodes a long non-coding RNA (lncRNA) that coats the X inactive chromosome in cis; thus, XIST is expressed on the X chromosome to be inactivated (Achilla et al., 2022). Xist is the main regulator of mammalian X inactivation. The *RNF12* gene acts as an upstream activator of Xist, and its protein product—the ubiquitin ligase that degrades the Xist repressor Rex1—has been shown to play a key role in the XX dose-dependent activation of Xist. Xist can aggregate specific proteins and interact with them to cover and silence an X chromosome (Wan et al., 2020). The proteins SAFA, LBR, and SHARP are essential for XCI. The hnRNP U/SAFA family of molecules controls Xist localization; LBR recruits inactive X chromosomes to the nuclear layer and alters the three-dimensional structure of DNA. LBR binds to Xist, allowing Xist and its silencing proteins to spread on the X chromosome to silence transcription, thereby remodeling the chromosome and making its genes less likely to be expressed. The “silencing” function is carried out by the protein SHARP, which excludes polymerase from DNA, thereby preventing transcription and gene expression (Wan et al., 2020). Then, chromatin modifications such as hypoacetylation of histone H4, accumulation of trimethylated histone H3 at K9 and K27, and accumulation of histone variant macro H2A on the inactive X chromosome promote gene silencing (Achilla et al., 2022).

X inactivation is a chromosome-wide mechanism, so most genes on the inactive X are repressed. However, it is estimated that up to 15%–20% of X-linked genes can escape inactivation; they remain expressed from both the active and inactive X. These escape genes are located throughout the X chromosome but predominate in the small regions of homology and pairing that persist on the sex chromosomes, called the pseudo-autosomal regions (PAR) (Berletch et al., 2011). Genes within the PAR are usually not subject to X inactivation since functional, equivalent alleles are present on the X and Y chromosomes. The non-pseudo autosomal genes that retain a Y-linked copy also often escape X inactivation and thus have two expressed alleles in both male and female somatic tissues (Berletch et al., 2011; Cáceres et al., 2025).

The study of X chromosome inactivation has significant implications, particularly for clinical management and genetic counselling of women carrying X-linked pathogenic variants (PV). Given that during embryogenesis the choice of which of the two X chromosomes is inactivated is random, there will be approximately the same probability of inactivating either paternal or maternal X chromosome. However, different inactivation ratios, defined as the proportion of cells expressing alleles from one or the other X chromosome, are observed (Kubota, 2001; Amos-Landgraf et al., 2006). The X-inactivation ratios of females can range from a highly skewed ratio of 0:100, where the same X chromosome is active for all cells, to a 50:50 ratio, where cells inactivate the paternal X chromosome and the other 50 inactivate the maternal chromosome. Non-random XCI or “skewed XCI”, has been arbitrarily defined as a pattern where 80% of more of the cells show a preferential inactivation of one of the X chromosomes. Skewed XCI patterns can be observed in over 25% of females. In unaffected females, the XCI ratio may be of no clinical importance; however, a highly skewed X inactivation can have important implications in women carrying X-linked chromosome diseases (Kubota, 2001; Amos-Landgraf et al., 2006; Ørstavik, 2006).

## 2 X-linked disorders

Approximately 867 protein-coding genes are located on the X chromosome (Migeon, 2020). PV that induce a complete loss of function in some of these genes can be lethal for both men and women. However, some less severe pathogenic variants occurring in less essential genes cause at least 533 X-linked diseases, which affect men more severely than women (Migeon, 2020). More than 130 X-linked genes have been associated with developmental disorders, and X-linked inheritance has been proposed as the underlying cause of the higher rate of developmental disorders observed in men. Most genes on the X chromosome are involved in the development of non-reproductive tissues, including the brain, blood, heart, liver, kidney, retina, skin, and teeth. There are numerous X-linked diseases, which exhibit high genotypic and phenotypic variability (Migeon, 2020; Martin et al., 2021).

Men and women are affected differently by X-linked diseases due to XCI (Migeon, 2020). This process has important implications for the effects of diseases caused by either X-linked PVs or numerical and/or structural changes in the X chromosome. Because of XCI, heterozygous women are mosaics for two cell populations that carry cells with either the normal or the altered-disease-causing allele-, activated (Migeon, 2020). For this reason, women are less susceptible to X-linked PVs (XPV) because the variant may not be expressed in all cells, whereas in men with XVPs the variant is expressed in all cells, conferring a more severe phenotype (Migeon, 2020). If the XPV is very severe, it can be lethal in men, which is why some X-linked diseases are only observed in women or in mosaic men, that is, with a combination of unaltered and altered cells (Franco and Ballabio, 2006).

Skewed XCI further increases the high genotypic and phenotypic variability of X-linked diseases. This type of inactivation can occur through both positive and negative selection mechanisms and can vary across tissues, developmental stages, and individuals, leading to variability in phenotypic severity. For some diseases, complete skewing of XCI has been observed, in which the altered X chromosome is inactivated in all cells and the normal X chromosome is expressed, or *vice versa*, thereby impacting the phenotype of patients or carriers of PV (Franco and Ballabio, 2006). For women with X-linked disorders, the study of XCI is relevant since a bias in inactivation could explain the manifestation of symptoms in women carrying “recessive” X-linked disorders, and could also explain the differences in penetrance and expressivity in patients with “dominant” disorders; therefore, studying the XCI pattern in women carrying X-linked PV is of great relevance in genotype-phenotype correlations to deepen the knowledge of these entities (Mundo-Ayala, 2008). X-linked diseases show a great plethora of clinical manifestations, and some of them show increased cancer risk.

## 3 X chromosome and cancer

Like any other autosomal chromosome, the X chromosome can be involved in cancer. However, its involvement in cancer development is peculiar given its unique characteristics, i.e., XCI and the dosage difference between males and females, which also affect its influence on cancer (Spatz et al., 2004). Human cancer exhibits a vast diversity of genetic and phenotypic characteristics that have been organized into the so-called “hallmarks of cancer,” defined as aberrantly acquired functional capabilities that underpin the mechanistic foundation of cancer, together with hallmark-enabling phenotypic characteristics, hallmark-conveying cells populating cancer microenvironments, and systemic interactions (Hanahan, 2026). In addressing how alterations of X-linked genes might contribute to cancer, the genetic hallmarks of cancer should be emphasized.

**FIGURE 1:**
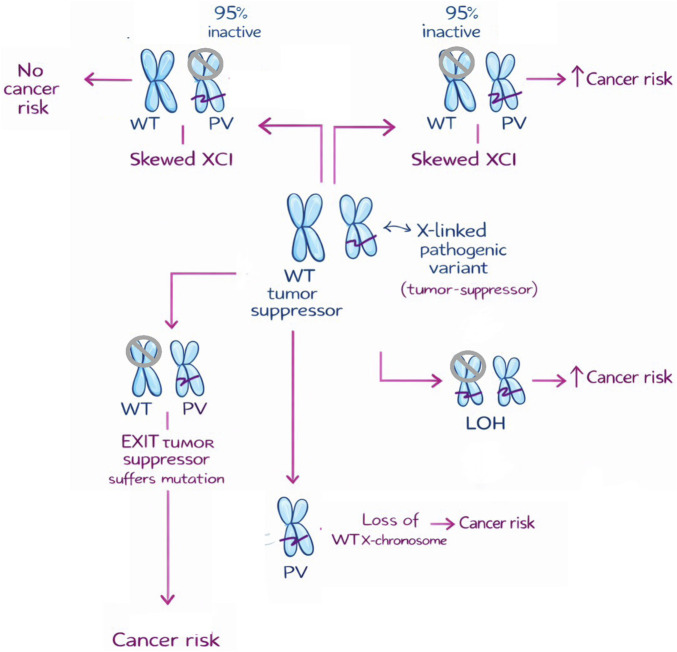
Possible mechanisms involved in oncogenesis driven by the X chromosome in females carrying a pathogenic variant in a tumor suppressor gene. If skewed XCI inactivates mostly the normal X chromosome, then the risk of cancer exists. On the contrary, if skewed inactivation silences the altered X chromosome, the wild-type allele of the tumor suppressor can be expressed from the active X chromosome, and there is no risk of cancer. If LOH occurs, with the wild-type allele lost and the altered allele duplicated, the risk of cancer exists. If aneuploidy occurs and the whole wild-type X chromosome is lost, then cancer can occur. If the normal chromosome is inactivated but the tumor suppressor is an EXIT gene (escapes inactivation), no cancer risk is observed. However, if that EXIT gene suffers an inactivating mutation, then cancer is a possibility. *XCI, X-chromosome inactivation; LOH, loss of heterozygosity; WT, wild-type tumor suppressor; PV, pathogenic variant in tumor suppressor; EXIT, escape from X-inactivation tumor suppressors. Chromosomes with cross circle represent an inactive chromosome. Purple line on the chromosome represents the pathogenic variant.

Nine hallmarks of cancer have been recognized: Sustaining proliferative signaling (activation of oncogenes), inactivating growth repressors (inactivating tumor suppressors), establishing replicative immortality, deregulating cellular metabolism, resisting programmed cell death, inducing or accessing vasculature, unlocking phenotypic plasticity, and evading immune destruction (Hanahan, 2026). Five enabling phenotypic characteristics that facilitate the acquisition of hallmark capabilities have been described: loss of genomic integrity, non-mutational epigenetic reprogramming, tumor-promoting inflammation, innervation, and polymorphic microbiomes. Seven hallmark-conveying cells populating cancer microenvironments have been recognized: Senescent cells, endothelial cells and pericytes, cancer-associated fibroblasts, neurons and their axonal projections, macrophages and neutrophils, and additional hallmark-facilitating immune cells (Hall et al., 2019). These cells are recruited and/or locally reprogrammed normal cells that are critical for the manifestation of cancer. Finally, interactions of cancer as a systemic disease are recognized, for example, with aging and obesity (Hanahan, 2026). As for autosomal genes, genetic alterations involving the X chromosome that can lead to cancer include gains and losses of chromosomes, genomic rearrangements, and mutations, which can activate oncogenes or inactivate tumor suppressors (Table 1) (Spatz et al., 2004; Dakal et al., 2024). However, given the peculiarities of the X chromosome, other cancer-related mechanisms are recognized. The X chromosome is involved in cancer through different mechanisms, including defective XCI, loss of heterozygosity, and germline and somatic alterations of X-linked genes (Figure 1).

**TABLE 1:** Tumor suppressor genes on the X chromosome.

Gene	Region	OMIM	Gene	Region	OMIM
*DDX3X*	Xp11.4	300160	*RBMX*	Xq26.3	300199
*DMD*	Xp21.2-p21.1	300377	*RPS6KA6*	Xq21.1	300303
*FOXP3*	Xp11.23	300292	*BTK*	Xq22.1	300300
*RBBP7*	Xp22.2	300825	*TCEAL7*	Xq22.2	300771
*BCORL1*	Xp11.4	300485	*EDA2R*	Xq12	300276
*SRPX*	Xp11.4	300187	*FOXO4*	Xq13.1	300033
*KDM6A*	Xp11.3	300128	*PHF6*	Xq26.2	300414
​	​	​	*BEX2*	Xq22.2	300691
​	​	​	*AMER1*	Xq11.2	300647
​	​	​	*RPL10*	Xq28	312173
​	​	​	*ZNF185*	Xq28	300381
​	​	​	*MAGEC3*	Xq27.2	300469
​	​	​	*TREX2*	Xq28	300370
​	​	​	*DUSP9*	Xq28	300134
​	​	​	*FHL1*	Xq26.3	300163
​	​	​	*FLNA*	Xq28	3000017
​	​	​	*GPC3*	Xq26.2	300037

Examples of X-linked tumor suppressor genes are given. Modified from (Dakal et al., 2024).

### 3.1 Defective XCI and cancer

Many studies have shown that the inactive X-chromosome is epigenetically unstable in cancer cells. Loss of the inactive X chromosome has been observed in many cancer types and is often accompanied by duplication of the active X chromosome, while there is evidence of decondensation and sporadic reactivation of the inactive X chromosome in cancer cell lines. Both mechanisms lead to overexpression of X-linked genes, which may be associated with cancer development and progression. Moreover, defects in XCI are common in many malignancies, including cancer. A high incidence of skewed X-chromosome inactivation has been reported in females with ovarian, breast, and lung cancer, indicating the presence of tumor-related genes on the X-chromosome (Achilla et al., 2022). It is reported that defective XCI is an additive factor to the cancer risk of XCI escape deregulation in women. Defective XCI of more than 10% is associated with an attributable risk of 40% across 12 cancers. Defective XCI increases triple-negative breast cancer frequency, decreases survival rates, and is reduced by chemotherapy treatment (Cáceres et al., 2025).

A direct causal relationship between lncRNA Xist and tumors has been demonstrated. Xist-led XCI processes silence hundreds of genes (including oncogenes); therefore, the loss of Xist expression promotes tumor development. Downregulation of Xist expression and loss of XCI are commonly observed in basal-like cancer, breast cancer susceptibility gene 1-null triple-negative breast cancer, and ovarian cancer cell lines. In addition, some patients with testicular germ cell tumors were found to have significantly higher levels of Xist demethylation (Wan et al., 2020). As Xist is critical for maintaining XCI, the deletion of Xist expression causes the inactivated X chromosome to be reactivated, triggering a series of unfavorable genome-wide changes that affect DNA replication, chromosome segregation, and cell cycle checkpoints. *Xist* is altered in a variety of human cancers; many cancer cell lines derived from female breast, cervical, and ovarian tumors show a loss of *Xist* expression (Wan et al., 2020). Although most of the genes on the inactive X chromosome are silenced, approximately 15% of genes escape inactivation and continue to be expressed in both X chromosomes. Many of the escapee genes are located in regions such as Xp11-22, Xq25—26, and Xq27-28, and it is proposed that these loci contain many tumor suppressor genes that can escape inactivation. These genes, named “escape from X-inactivation tumor suppressors (EXITS), are often located outside the Xist RNA coating region (Wan et al., 2020; Dakal et al., 2024).

*BRCA1* is an autosomal tumor suppressor gene located at 17q21 that plays a role in DNA repair, and its mutations are involved in the development of ovarian and breast cancer. Interestingly, *BRCA1* has a role in XIST RNA accumulation on the inactive X chromosome. Evidence suggests that intact *BRCA1* functioning is required for the maintenance of XIST localization to the inactivated X chromosome. Defects in the X chromosome might contribute to oncogenesis caused by the mutation (Spatz et al., 2004).

### 3.2 Loss of heterozygosity (LOH) of X-linked genes

The common genetic events that cause cancer—oncogene activation and tumor-suppressor inactivation—are expected to produce different results when they affect genes that are carried on the X chromosome. Gain-of-function mutations leading to oncogene activation—which acts dominantly when it affects autosomal genes—might remain silent if they occur on the inactivated allele of an X-linked gene. By contrast, tumor-suppressor gene inactivation by loss of a single allele (loss of function), which is recessive when it affects autosomal genes, might become dominant when the other allele is functionally silenced due to X-chromosome inactivation. X-chromosome inactivation, therefore, represents an operational LOH (Spatz et al., 2004).

Loss of heterozygosity (LOH) of X-linked genes is also a common occurrence in carcinogenesis. Among the various types of genomic mutations, LOH events are the most common and affect a larger portion of the genome. They typically arise from recombination-mediated repair of double-strand breaks (DSBs) or from lesions that are processed into DSBs. LOH events are critical drivers of genetic diversity, enabling rapid phenotypic variation and contributing to tumorigenesis (Dutta and Schacherer, 2025). LOH typically occurs through the exchange of genetic material between homologous chromosomes, primarily via mitotic recombination, as well as other mechanisms. LOH events can be categorized into two types: copy-loss LOH (CLLOH) and copy-neutral LOH (CN-LOH). In CL-LOH, a cell that was initially heterozygous at a particular locus loses one of its two alleles due to the deletion of one allele. In contrast, CN-LOH involves the loss of one allele, but instead of deletion, the remaining allele is duplicated to maintain the copy number (Dutta and Schacherer, 2025).

LOH is generally associated with tumor suppressor genes. In a female cell, a tumor suppressor gene is in an inactive state if it is on the inactive X; thus, when the other tumor suppressor allele on the active X chromosome is mutated or deleted, the malignant state is no longer inhibited, resulting in a decrease in the level of tumor suppressor function during tumor development and progression, driving cells to cancer transformation (Figure 1). LOH on the active X chromosome may result in the complete loss of tumor suppressor function in these X-linked genes, making the individual susceptible to cancer formation; this contrasts with biallelic inactivation of autosomal tumor suppressor genes, whose expression levels remain sufficient to function in human cancers. X-linked genes such as *ATRX*, *KDM6A*, *CNKSR2*, *DDX3X*, *KDM5C*, and *MAGEC3* can escape XCI and thus play a role in inhibiting tumorigenesis. However, these genes exhibit higher mutation frequencies in cancer and are presumed to be important candidate EXITS genes (Wan et al., 2020; Berletch et al., 2011; Dunford et al., 2017).

X-linked LOH has been reported in several cancers. It has been reported that recurrent loss of Xq25 occurs in 52% of female breast adenocarcinomas. Also, X-linked LOH is seen in approximately 40% of sporadic ovarian cancer, frequently affecting the Xq25-26 region. In renal-cell carcinoma, X-linked LOH is associated with aggressiveness and prognosis. In addition, LOH involving the X chromosome has been reported in all metastatic gastric carcinoid tumors and in 60% of infiltrative metastatic gastroenteropancreatic tumors (Spatz et al., 2004). *BRCA1* mutations have also been associated with X-chromosome LOH. For example, LOH at Xp22.2–22.3 has been reported to be associated with germline *BRCA1* mutations in ovarian cancer. LOH in this region is twice as frequent in carriers of *BRCA1* germline mutations and predominantly affects the active allele, supporting the hypothesis of a tumor-suppressor gene locus at Xp22.2–22.3 and implicating LOH of this gene in *BRCA1*-linked carcinogenesis (Spatz et al., 2004; Roncuzzi et al., 2002).

### 3.3 X-linked germline and somatic alterations

Like other diseases, cancer can be initiated by a single genetic event occurring either in somatic cells or in germ cells, leading to sporadic cancers or hereditary cancers, respectively (Spatz et al., 2004). Somatic and germline alterations of genes on sex chromosomes may be involved in tumorigenesis. The X-chromosome carries a significant number of oncogenes and tumor-suppressor genes (Dakal et al., 2024). Mutations or dysregulation of these genes might be putative mechanisms for cancer development. Several X-linked genes show germline genetic variants that increase cancer predisposition. Achilla et al. reviewed genetic polymorphisms on the X chromosome involved in cancer susceptibility (Achilla et al., 2022).

As for autosomal genes, genetic alterations involving the X chromosome that can lead to cancer include gains and losses of chromosomes, genomic rearrangements, and mutations, which can lead to activation of oncogenes or loss of function of tumor suppressors. Both of the main types of genetic alterations that lead to cancer—tumor-suppressor inactivation and oncogene activation—act dominantly when they affect the single active copy of an X-linked gene. The same alterations remain silent when they affect the inactivated X chromosome in female cells. Translocations involving regions of the X chromosome have distinct outcomes regarding their ability to cause cancer. Events involving the relocation of regions of the inactive X chromosome to an autosome can reactivate previously silent X-linked genes, with potential oncogenic effects. Conversely, loss of expression of an autosomal tumor suppressor can result from translocation to the inactive X chromosome (Spatz et al., 2004).

### 3.4 Numerical X chromosome alterations and cancer

All kinds of somatic X-chromosome gains—gains of whole chromosomes or chromosome arms, and gene amplifications—have been observed in many types of solid and hematopoietic tumors. Loss of the X chromosome is the most common clonal somatic alteration in leukocytes of females; however, it has been associated with the risk of myeloid and lymphoid leukemias (Liu et al., 2024). Whole chromosome gains are seen in prostate carcinomas, and they have an important role in the progression from chronic phase to blast crisis in chronic neutrophilic leukemia. In the case of germline X chromosome gains, achievement of a correct gene dosage is expected through XCI; however, men with XXY syndrome and XX male syndrome are known risk factors for male breast cancer (Spatz et al., 2004).

## 4 X-linked diseases and cancer

There are more than 500 described X-linked disorders, many of them showing a particularly high risk of cancer development (Migeon, 2020). Many X-linked disorders show PV in genes that also play a role in cancer development, particularly in tumor suppressor genes. Table 2 shows X-linked disorders and their cancer risk. All the literature in this review was searched on PubMed and Web of science databases, the terms: “X-linked and cancer or malignancy/malignant”; X chromosome and cancer or malignancy/malignant”; “X-linked disorder and cancer risk”; “X inactivation and cancer or malignancy/malignant”; and “X-dimorphism and cancer” were used as an initial search. Original and review articles of the last 5 years were selected. Abstracts were examined and selected if they were within the scope of this review. For the construction of Table 2, based on the X-linked disorder list offered by Migeon et al., 2020 each gene was searched on the *Cancer Genetics Web*. X-linked disorders with genes involved in cancer were selected. Gene selection and X-linked disease annotation were based on the Online Mendelian Inheritance in Man (OMIM) database (omim.org). Cancer associations were investigated hierarchically across three sources: the NCI Genes and Cancer database (cancer.gov), the COSMIC Cancer Gene Census (cancer.sanger.ac.uk/census), and, for genes absent from both, a PubMed/MEDLINE search using the strategy [gene symbol] AND (cancer OR carcinoma OR tumor OR leukemia OR lymphoma OR sarcoma OR malignancy), supplemented by CancerMine (bionlp.bcgsc.ca/cancermine). Original research articles and systematic reviews were included; editorials, conference abstracts, and preprints were excluded. With the selected X-linked disorders, another search was performed in PubMed using the name of each X-linked disorder, together with the term “cancer”. The papers were analyzed looking for cancer risk in X-linked disorder patients or the involvement of the X-linked gene in any type of cancer.

**TABLE 2:** X-linked disorders and cancer risk.

Gene #OMIM	X-linked disease	Cancer	Genes and cancer web	Mechanism	References
*ATRX* 300032	Alpha-thalassemia/mental retardation syndrome, X-linked (ATR-X syndrome)	Osteosarcoma	Astrocytoma (childhood); Brain and CNS tumors; Brain stem glioma (childhood); Brain tumors (childhood); Neuroblastoma; Pancreatic cancer; Von Hippel-Lindau disease [cancer.gov]	Alternative lengthening of telomeres (ALT) pathway, a telomerase-independent mechanism that confers replicative immortality on cancer cells	Yuan et al. (2024), Wang et al. (2025)
*UBA1* 314370	VEXAS syndrome (vacuoles, E1 enzyme, X-linked, autoinflammatory, somatic)	Myelodysplastic syndrome, multiple myeloma	Lung cancer; Hematological cancers [cancer.gov]	UBA1 orchestrates DNA damage response; partial loss-of-function mutations disrupt ubiquitin pathway and proteasomal degradation	Sakuma et al. (2024), Gavioli et al. (2026)
*FOXP3* 300292	Immune dysregulation, polyendocrinopathy, enteropathy, X-linked (IPEX) syndrome	Breast cancer; Colorectal cancer; Melanoma; Prostate cancer; Skin cancer [cancer.gov]	FOXP3 is a tumor suppressor essential for self-tolerance maintenance, Treg cell development/function, and prevention of autoimmune disorders and tumorigenesis	One case of lymphoma reported	Liu et al. (2013)
*FANC B* 300515	Fanconi anemia, complementation group B (X-linked FA); VACTERL-H association	Acute myeloid leukemia; Squamous cell carcinomas of head and neck; Anogenital squamous cell carcinomas	Multiple cancer types (general predisposition) [cancer.gov]	Defective DNA interstrand crosslink repair via the Fanconi anemia-BRCA pathway; leads to genomic instability and chromosomal aberrations	Choi and Jung (2025), Chen et al. (2014)
*WAS* 300392	Wiskott-Aldrich syndrome (WAS)	Hematolymphoid malignancies	Not listed in cancer.gov Genes and Cancer database COSMIC CGC Tier 2: WAS tumor suppressor in hematologic malignancies CancerMine/Literature: ALCL, CML, B-cell NHL, T-cell NHL	Alterations within cell signaling and cytoskeletal remodeling needed for cancer cells to execute malignant activities including aberrant proliferation, activation, migration and invasion	Hosahalli Vasanna et al. (2021), Tian et al. (2025)
*PHF6* 300414	Börjeson-Forssman-Lehmann syndrome (BFLS)	Acute Myeloid Leukaemia, T cell lymphoblastic leukemia	Acute myeloid leukemia [cancer.gov]	PHF6 is a chromatin regulator with tumor suppressor activity. PHF6 is a somatic driver of T cell leukemia which cooperates with NOTCH1 activation	Kurzer and Weinberg (2021), Jain et al. (2023)
*BTK* 300300	X-linked agammaglobulinemia (Bruton disease)	Non-Hodgkin lymphoma; Gastric adenocarcinoma; Colorectal cancer	B lymphoid malignancies [cancer.gov]	BTK acts as a tumor suppressor playing a crucial role in B-cell differentiation, affecting proliferation and apoptosis; somatic BTK alterations disrupt these functions	Brosens et al. (2008)
*DKC1* 300126	Dyskeratosis congenita, X-linked (Zinsser-Cole-Engman syndrome)	Head and neck squamous cell carcinoma; Acute myeloid leukemia; Non-Hodgkin lymphoma	Breast cancer; Colorectal cancer; Lung cancer; Pituitary tumors; Prostate cancer; Skin cancer [cancer.gov]	Telomere shortening and genomic instability; impaired ribosome biogenesis; chromosomal instability and telomere dysfunction predispose to malignant transformation	Niewisch and Savage (2019)
*GLA* 300644	Fabry disease (alpha-galactosidase A deficiency)	Marginally reduced overall cancer rate; possibly increased rates of melanoma, urological malignancies and meningiomas	Glioma [cancer.gov]	Stimulation of oxidative stress, inflammation and angiogenesis by sphingolipid (Gb3) accumulation; GLA expression influences prognosis by modulating tumor-infiltrating immune cells	Bird et al. (2017), Rossi et al. (2019)
*G6PD* 305900	Glucose-6-phosphate dehydrogenase deficiency	Reduced cancer of endodermal origin; not of ectodermal/mesodermal origin	Chronic myelogenous leukemia; Leukemia; Myeloproliferative disorders; Parathyroid cancer; Thyroid cancer; Wilms tumor [cancer.gov]	G6PD overexpression influences DNA synthesis, DNA repair, cell cycle regulation, redox equilibrium, proliferation, EMT, invasion and metastasis	Song et al. (2022), Pes et al. (2019)
*L1CAM* 308840	X-linked hydrocephalus (HYCX); MASA syndrome; Spastic paraplegia 1 (SPG1)	No increased cancer risk associated with germline disease	Breast cancer; Endometrial cancer; Melanoma; Osteosarcoma; Ovarian cancer; Pancreatic cancer; Skin cancer; Stomach cancer [cancer.gov]	Activation of the NF-κB (p65) pathway promotes cell proliferation and contributes to drug resistance; L1CAM overexpression promotes tumor invasion and metastasis	Schrander-Stumpel et al. (1995), Kurosu et al. (2026)
*KDM6A* 300128	Kabuki syndrome 2 (KDM6A loss-of-function)	Some cases of cancer	Breast cancer; Kidney cancer; Medulloblastoma; Pancreatic cancer; Prostate cancer [cancer.gov]	Epigenetic deregulation: KDM6A is an H3K27me3 demethylase; loss leads to repression of tumor suppressor genes; transcriptional reprogramming facilitates oncogenesis	Roma et al. (2015), Teranishi et al. (2018)
*HPRT* 308000	Lesch-Nyhan syndrome; Kelly-Seegmiller syndrome	No clearly increased cancer risk	Bladder cancer; Cervical cancer; Skin cancer; Wilms tumor [cancer.gov]	Alteration of purine metabolism leading to altered nucleotide pools; genomic instability via disrupted DNA synthesis and repair	Shields et al. (2018), Lu et al. (2024)
*TLR7* 300365	Severe systemic lupus erythematosus (TLR7 gain-of-function)	Gastric/esophageal/colon/anal/hepatobiliary/pancreatic cancers; Hodgkin and non-Hodgkin lymphoma; Leukemia; Multiple myeloma; Lung; Larynx; Cervical; Vaginal/vulvar; Renal; Bladder; Skin; Thyroid cancers	Bladder cancer; Cervical cancer; Skin cancer; Wilms tumor [cancer.gov]	Activates NF-κB and MAPK cascades (via MyD88); sustained production of pro-inflammatory cytokines; enhances PI3K/AKT and ERK signaling promoting a tumor-permissive inflammatory microenvironment	Zhang et al. (2022)
*XIAP* 300079	Lymphoproliferative syndrome 2, X-linked (XLP2)	No increased cancer risk associated with the X-linked disease	Bladder cancer; Breast cancer; Colorectal cancer; Liver cancer; Ovarian cancer; Pancreatic cancer; Stomach cancer [cancer.gov]	Blocks caspase activation; inhibits mitochondrial apoptosis pathway; activates NF-κB pathway; XIAP overexpression in cancer cells confers resistance to apoptosis	Holcik et al. (2001)
*SH2D1A* 300490	Lymphoproliferative syndrome, X-linked 1 (XLP1; Duncan disease)	Lymphoma, most often B-cell non-Hodgkin lymphoma	Hodgkin lymphoma; Non-Hodgkin lymphoma [cancer.gov]	Failure of viral immune containment (failure to control EBV infection); lymphoproliferative instability promotes malignant transformation	Nichols et al. (1998)
*MED12* 300188	Lujan-Fryns syndrome; FG syndrome (Opitz-Kaveggia); Ohdo syndrome	Prostate cancer (somatic MED12 mutations)	Adrenocortical cancer; Breast cancer; Prostate cancer; Soft tissue sarcoma; Uterine sarcoma [cancer.gov]	Alteration of transcriptional regulation mediated by the Mediator complex; somatic MED12 mutations disrupt CDK8 signaling	Gonzalez et al. (2022)
*FLN* 300017	Oropalatodigital syndrome types 1 and 2; Melnick-Needles syndrome; Frontometaphyseal dysplasia 1	No increased cancer risk associated with the X-linked disease	Acute myeloid leukemia; Bladder cancer; Breast cancer; Melanoma; Prostate cancer [cancer.gov]	Cytoskeletal remodeling via filamin A (FLNA) overexpression; regulates cell migration, invasion and survival signaling pathways	Savoy and Ghosh (2013)
*ATP7A* 300011	Menkes disease; Occipital horn syndrome; X-linked spinal muscular atrophy type 3	No increased cancer risk associated with the X-linked disease	Breast cancer; Colorectal cancer; Lung cancer (NSCLC); Ovarian cancer; Skin cancer [cancer.gov]	Copper transport-mediated angiogenesis and chemoresistance; ATP7A modulates cisplatin efflux and copper-dependent antiangiogenic factors	Li et al. (2018), Ramani and Parayil Sankaran (2025), Zhu et al. (2017)
*BCOR* 300485	Microphthalmia, syndromic 2 (MCOPS2); Oculofaciocardiodental syndrome (OFCD)	No increased cancer risk associated with the X-linked disease	Acute myeloid leukemia; Bone cancer (primary); Endometrial cancer; Ewing sarcoma; Kidney cancer; Skin cancer; Soft tissue sarcoma [cancer.gov]	Epigenetic derepression via PRC1.1 complex dysfunction; BCOR loss disrupts H2AK119 ubiquitination and Polycomb-mediated gene silencing	Astolfi et al. (2019), Tara et al. (2018)
*HCCS* 300056	Microphthalmia, syndromic 7 (MCOPS7); MIDAS syndrome	No increased cancer risk associated with the X-linked disease	Liver cancer [cancer.gov]	Impaired mitochondrial apoptosis regulation; HCCS encodes holocytochrome c-type synthase required for cytochrome c assembly and electron transport chain	Indrieri et al. (2013)
*MECP2* 300005	Rett syndrome (females); MECP2 duplication syndrome (males); Sick sinus syndrome 2	Lower risk of cancer (Rett syndrome); No increased risk (MECP2 duplication)	Breast cancer; Prostate cancer [cancer.gov]	Regulation of genes linked to tumorigenesis; alterations in reading and writing of epigenetic marks linked to tumor progression; MECP2 modulates DNA methylation and chromatin organization	Awortwe et al. (2024), Yilmaz et al. (2014)
*GPC3* 300037	Simpson-Golabi-Behmel syndrome type 1 (SGBS1)	Hepatoblastoma; Wilms tumor	Wilms tumor; Hepatoblastoma; Liver cancer; Lung cancer; Breast cancer; Thyroid cancer [cancer.gov]	Dysregulation of apoptosis signaling and cell migration; GPC3 modulates Wnt, Hedgehog, and FGF pathways; acts as co-receptor for growth factors	Akkermans et al. (2022), Nisbet et al. (1993)
*GPC4* 300168	Keipert syndrome	No increased cancer risk associated with the X-linked disease	Glioblastoma; Non-small cell lung adenocarcinoma; Pancreatic cancer; Breast cancer; Colorectal cancer [cancer.gov]	GPC4 undergoes downregulation in metastatic tumors; overexpression of GPC4 induces decreased tumorigenicity; modulates FGF and Wnt signaling	Hansson et al. (2024)
*IL2RG* 308380	X-linked severe combined immunodeficiency (SCIDX1; CIDX1)	T-cell leukemia (post-gene therapy insertional mutagenesis)	Acute myeloid leukemia; Breast cancer; Chronic myelogenous leukemia; Leukemia; Lung cancer [cancer.gov]	Encodes the common gamma chain (γc), essential for cytokine signaling (IL-2, IL-4, IL-7, IL-9, IL-15, IL-21); loss leads to absence of T, B and NK cells; impaired immune surveillance allows tumor escape	Kim et al. (2025), Cavazzana et al. (2016)
*KDM5CI/JARIDC* 314690	XLID Claes-Jensen	Not established	Not listed in cancer.gov Genes and Cancer database COSMIC CGC Tier 2: KDM5C listed in renal cell carcinoma and other solid tumors Literature: Kidney cancer (clear cell renal cell carcinoma —Pancreatic cancer; Bladder [COSMIC CGC/Literature]	Somatic pathogenic variants are associated with high angiogenesis and AMPK/Fatty acid oxidation gene expression	Müller et al. (2025), Sellner et al. (2023)
*AR* 313700	Androgen insensitivity syndrome (complete/partial); Kennedy disease (SBMA — spinal and bulbar muscular atrophy)	No clearly increased cancer risk from the germline X-linked disease itself (AIS/Kennedy)	Not listed in cancer.gov Genes and Cancer database for germline disease COSMIC CGC Tier 1: AR classified as oncogene Literature: Prostate cancer Breast cancer —Bladder cancer — Multiple myeloma, Melanoma, Rhabdomyosarcoma, Pancreatic cancer [COSMIC CGC/Literature]	AR transcriptionally activates genes governing proliferation (cyclin D1, c-MYC) and survival	Lanciotti et al. (2019)
*CD40L* 300386	Immunodeficiency with hyper-IgM type 1 (HIGM1); Immunodeficiency 3	Hepatocellular carcinoma and bile duct carcinomas (in HIGM1 context)	Breast cancer; Lung cancer; Colorectal cancer; Prostate cancer; Melanoma; Bladder cancer; Ovarian cancer; Pancreatic cancer; Liver cancer [cancer.gov]	Immune modulation: CD40L activates CD40 on antigen-presenting cells and B cells; loss impairs anti-tumor immunity; CD40^−^CD40L axis also involved in direct tumor cell signaling	Pazoki et al. (2024), Erdos et al. (2008)
*COL4A5* 303630	Alport syndrome, X-linked (XLAS)	No increased cancer risk associated with the X-linked disease	Esophageal cancer [cancer.gov]	Somatic loss-of-function mutations in collagen genes (including COL4A5) reported in various solid tumors; collagen IV is a key basement membrane component, and its disruption facilitates tumor invasion	Brodsky et al. (2022), Heidet et al. (1998)

As expected, not all the X-linked disorders show a high risk of cancer (Table 2), even though the responsible gene of the disease is also involved in tumorigenesis. These could be explained by different pathogenic variants and by the fact that in X-linked disorders the gene alteration is germinal, whereas in sporadic cancers the alterations are somatic. Also, factors such as XCI, play an important role in determining cancer risk in patients with an X-linked disease. Several X-linked disorders show complete skewed XCI, and this phenomenon could further increase cancer development. Table 2 describes the cancer risk associated with X-linked disorders, including the gene causing the X-linked disease (germline mutations) and whether that gene also participates in sporadic cancers (somatic mutations). The mechanism of the carcinogenic role of the genes is also described. Four representative X-linked disorders and their associated cancer risks are discussed below.

ATRX syndrome includes five main typical clinical manifestations: facial dysmorphism, hypotonia, skeletal, genitourinary system, and hematopoietic abnormalities. The *ATRX* gene was first identified as a pathogenic gene for a rare hereditary disease that causes intellectual disability and α-thalassemia. ATRX belongs to the switch/sucrose nonfermentable (SWI-SNF) protein clan and functions as a chromatin remodeler, many of which can slide, modify, or remove histones (Yuan et al., 2024). ATRX syndrome is usually asymptomatic in women due to significant skewing of X chromosome inactivation, while the symptoms are more obvious in men due to the absence of alleles. In approximately 10%–15% of tumors, telomere length is maintained via alternative lengthening of telomeres (ALT) rather than telomerase activation. Given the crucial role of telomere length maintenance in cell perpetuation and tumorigenesis, the strong correlation between *ATRX* deficiency and ALT activation suggests that ATRX may suppress tumors by directly inhibiting ALT. *ATRX* is proposed as an EXIT tumor suppressor gene, with mutations identified in many cancers such as neuroblastoma, pancreatic neuroendocrine tumors, and osteosarcoma. Although *ATRX* mutations are seen in diverse types of cancer, patients with ATRX syndrome are described to have a risk of osteosarcoma (Wan et al., 2020; Yuan et al., 2024).

Another X-linked disorder carrying an EXIT tumor suppressor gene alteration is Kabuki syndrome 2. This disease is caused by pathogenic variants in the *KDM6A* gene, which is a histone demethylase that escapes X chromosome inactivation and is ubiquitously expressed. It specifically mediates the removal of di- and trimethylation marks on histone H3 Lys27, and its loss or inactivation has often been correlated with the onset of a broad spectrum of congenital anomalies, particularly type 2 Kabuki Syndrome. Notably, *KDM6A* somatic mutations can be found in multiple cancer types, including B-cell lymphoma, bladder urothelial carcinoma, head and neck squamous cell carcinoma, pancreatic adenocarcinoma, lung squamous cell carcinoma (LUSC), and kidney renal papillary cell carcinoma. However, their contribution to oncogenesis and tumor progression is still poorly characterized (Biagini et al., 2022). In contrast, type 2 Kabuki syndrome is not considered a cancer predisposition syndrome, and only some cases of cancer have been reported in these patients (Roma et al., 2015).

The *FANC B* gene is responsible for X-linked Fanconi anemia, which is a bone marrow failure syndrome with an increased incidence of cancers due to DNA repair deficiency. Mutations in at least 23 genes have been implicated in FA-defective DNA repair, and clinical manifestations may vary significantly. FA implicates a dramatically increased risk for both hematologic and solid malignancies, thus rendering patients vulnerable to early-onset, aggressive cancers. The deficient FA pathway not only plays a crucial role in tumorigenesis in FA but also contributes to tumorigenesis in sporadic cancer, such as head and neck cancer (Choi and Jung, 2025).

*UBA1* loss-of-function mutations cause VEXAS syndrome, a hemato-inflammatory disorder characterized by inflammation, cytopenias, thrombotic tendencies, clonality, and hematological malignancies, such as myelodysplastic neoplasms and multiple myeloma. Notably, despite the increased risk of acute myeloid leukemia (AML) in MDS patients, progression to AML is extremely rare in patients with VEXAS-MDS. Although the association between *UBA1* mutations and cancer remains uncertain, there is evidence linking non-M41 (not affecting codon M41) *UBA1* mutations to various cancers (Sakuma et al., 2024). *UBA1* is a known orchestrator of DNA damage response, and coinciding with the discovery of VEXAS syndrome, *UBA1* mutations were implicated as potential key factors in the development of lung cancer among non-smokers. The patients were all females. None of them harbored the M41 mutations (codon M41), and instead, frameshift, nonsense, and non-M41 missense mutations. Somatic non-M41 variants are detected in various hematologic neoplasms, including lymphoid malignancies. The pathogenicity of the variants has not been confirmed, but different degrees of loss-of-function mutations of *UBA1* may have oncogenic potential (Sakuma et al., 2024).

## 5 Sexual dimorphism in cancer

The occurrence, progression, molecular phenotype, and treatment response to cancer can be biased depending on sex. Sex affects the incidence and outcome of most non-reproductive cancer types (Corchete Sanchez and Rheinbay, 2026). Compared to women, men show higher incidences of certain cancers, such as colorectal, lung, renal, esophageal, and other cancers (Feng et al., 2024). Malignant tumors such as esophageal cancer are diagnosed more frequently in men and are characterized by worse prognoses and higher mortality. These differences are thought to be linked to differences in immune response, hormone levels, and immune cell functions (Achilla et al., 2022; Feng et al., 2024). Also, sex differences are attributed to genetic variations between XX and XY individuals. Although these differences are partially mitigated through XCI, genes escaping from XCI may explain sexual dimorphism in cancer. Another potential difference underlying sex-specific manifestations of diseases are miRNAs. The X chromosome encodes 118 miRNAs while the Y chromosome encodes only 4. The presence and activity of X-linked regulatory miRNAs underscore significant sex disparities in the occurrence and progression of cancers.

The involvement of the X chromosome in cancer is also seen in men. The reduction in cancer incidence in women could be explained by the biallelic expression of the EXITS genes (Dunford et al., 2017). Because males have only one X chromosome, inactivation of a single allele of a tumor suppressor on the X chromosome could allow cancer to occur, in contrast to women, who could have the other allele of the tumor suppressor activated if it escapes inactivation; thus, retaining suppression of cancer activity. Six EXIT genes have been reported to evade XCI, leading to sex differences in cancer risk. Compared to women, mutation in the EXITS gene is more common in men with *ATRX, CNKSR2, DDX3X, KDM5C, KDM6A*, and *MAGEC3*, with a higher frequency of mutation loss. In addition, the environmental and hormonal factors associated with sex specific differences in cancer may interact with the EXITs loci or their products to modulate cancer risk (Wan et al., 2020; Dunford et al., 2017; Rubin et al., 2020).

Besides the X chromosome involvement in cancer, the Y chromosome participation is also recognized. Variation in the Y chromosome can affect both the quantity and function of immune cells, thereby influencing disease resistance. Loss of Y chromosome (LOY) refers to the partial or complete absence of the Y chromosome in male cells. LOY leads to the simultaneous loss of multiple presumed tumor suppressor genes (Corchete Sanchez and Rheinbay, 2026). This event is commonly observed in blood cells and progressively accumulates with age. LOY is closely associated with a shortened lifespan and increased incidence and mortality of various diseases, including cancer. LOY has been reported in cancers, including colorectal, prostate, bladder, pancreatic, and hematopoietic cancer (Feng et al., 2024).

## 6 Discussion

More than 500 X-linked diseases have been reported, and the severity of the clinical manifestations depends in part on XCI. The choice of the X chromosome to be inactivated can thus determine whether an individual will be healthy or affected by an X-linked disease, as well as the degree of affection (Sun et al., 2022). In addition, this selection could also influence the risk of an X-linked carrier to develop cancer, which is a common event in some X-linked diseases. Studying cancer risk and its association with XCI in individuals with X-linked diseases is complex, as XCI can vary with age and across tissues. In addition, skewed XCI does not always explain the phenotypic variability observed in X-linked disorders. In fact, the relationship between skewed XCI and phenotype in these individuals has been controversial in some disorders (Ørstavik, 2006; Sun et al., 2022).

Additionally, the method used to study XCI can add complexity to the associations of X cancer risk in individuals with X-linked diseases, as different methods may yield different information; the most accepted method is PCR (polymerase chain reaction) of polymorphic (CAG)n repeat in the first exon of the androgen receptor gene (HUMARA). After digestion of the DNA with the methylation-sensitive enzyme Hpa II, a PCR product is obtained from the inactive X chromosome only. However, other methods are used and may result in disparities compared with HUMARA PCR (Fadra et al., 2024). In addition, most studies are conducted in easily accessible cells, such as leukocytes, urinary, and buccal cells, rather than in tissues from affected organs or tissues prone to cancer development (Sun et al., 2022). Thus, the clinical significance of studying easily accessible cells to assess XCI should be taken with caution, as this may vary among tissues (Ørstavik, 2006). XCI not only varies across tissues but also varies with age, further complicating the study of XCI and its association with cancer. It is proposed that age-related skewing of X inactivation involves both stochastic and genetic events (Ørstavik, 2006). In a large methylome study, X reactivation was detectable in blood and increased with age and cancer diagnosis (Cáceres et al., 2025). In fact, XCI maintenance is proposed as a hallmark of female cancer in general. An additional risk factor for women is 1-allele methylation of escapees, associated with a higher loss of function mutations. In males, the silencing of escapees is associated with mosaic LOY, and cancer risk. In male cancer, mosaic LOY is linked to loss-of-function mutations in escapees with homologous copies on the Y chromosome (Cáceres et al., 2025). These highlight the importance of defective XCI in cancer risk, which needs further research to understand the precise mechanisms leading to cancer and to identify potential diagnostic or prognostic biomarkers. A possible mechanism of cancer risk from defective XCI has been proposed to involve alterations in the immune response (Cáceres et al., 2025).

Also, genes escaping from XCI further complicate the study of cancer risk in individuals with X-linked disorders. Patterns of gene escape can vary across different individuals, ages, and cell types. Some genes are found to escape in most cell types, while others are highly variable depending on cell origin (Sun et al., 2022). XCI escape genes are particularly prevalent in autoimmune disorders, such as systemic lupus erythematosus. Escape from XCI is common in immune cells and is subject to dynamic regulation, potentially increasing the susceptibility of females to autoimmune disorders. In contrast, in the cancer scenario, the escape of tumor suppressor genes from XCI could be protective in females, unless loss of mutations occurs in those escapees (Feng et al., 2024).

Although several X-linked disorders have been associated with a higher cancer risk, the mechanisms are complex and not fully understood. The exploration of these patients is somehow challenging, as for some of these disorders, not so many patients are reported, and some of them could die before developing cancer. Some of the X-linked disorders are the result of pathogenic variants in genes that have a role in cancer, which may explain the cancer risk in these patients. As expected, not all the X-linked disorders show a high risk of cancer, even though the responsible gene of the disease is also involved in tumorigenesis. These could be explained by different pathogenic variants and by the fact that in X-linked disorders the gene alteration is germinal, whereas in sporadic cancers the alterations are somatic. Also, factors discussed, such as XCI, play an important role in determining cancer risk in patients with an X-linked disease. Several X-linked disorders show complete skewed XCI, and this phenomenon could further increase cancer development. Many X-linked disorders show immune alterations, which have been established as a mechanism for cancer risk; however, more research is needed to determine the cancer risk mechanism in each particular disorder.

The clinical manifestations of X-linked diseases and their potential cancer risk can be influenced by the choice of the X chromosome to be inactivated. The dynamics of human X dosage compensation initiation remain debated, and further research is needed to fully understand this critical process (Furlan and Galupa, 2022). In humans, two models have been proposed to describe the initiation of XCI: the X-dampening and the direct X inactivation. The dampening model remains controversial; it considers a progressive increase in biallelic XIST expression, leading to a gradual downregulation of X-linked genes from morula to blastocyst, comparable to an “absence of choice”. The selection between two equal downregulated X chromosomes to determine which one to become inactive remains largely unknown (Alfeghaly et al., 2024). In contrast, the second model proposes direct X-chromosome inactivation (Furlan and Galupa, 2022).

Some factors could influence the choice of the X chromosome to be inactivated. Although in theory, both X chromosomes during random XCI have the same probability of being silenced, deviations from a 50:50 inactivation pattern are observed. This skewing can occur stochastically, due to non-random choice at the onset of XCI (primary choice), or as a result of selection for or against cells carrying one specific active or inactive X chromosome (secondary choice). In primary skewing, any variant in genes involved in XCI could influence the choice of the X chromosome to be inactivated. Secondary choice is particularly common among individuals with X-linked diseases. This secondary choice may give a survival advantage to cells with a particular X chromosome, either the affected or the normal (Furlan and Galupa, 2022). This same situation could apply to cancer, in which the secondary choice could confer a selective advantage to cells carrying a particular oncogenic X-linked variants.

## 7 Conclusion

The X chromosome plays a crucial role in cancer development, and thus its study, together with the processes associated with it, such as XCI, is highly relevant, as many gaps remain. Research in this arena could provide insights into the mechanisms underlying the X chromosome’s role in cancer and the identification of potential therapeutic targets or diagnostic/prognostic biomarkers. In addition, sex differences in cancer risk are of great relevance and warrant further investigation as they explain the different incidences of cancer risk among males and females. Moreover, X chromosomal alterations give rise to hundreds of X-linked diseases, many of which show a particular cancer risk. The study of cancer risk in individuals with X-linked diseases is highly complicated, given the great heterogeneity of X-linked pathogenic variants (many of which are cancer-related), the defects on XCI, and the escape from XCI, which vary across tissues and with age. The study of cancer risk in X-linked disorders deserves attention to fully delineate the mechanisms and to improve the counseling and management of these patients.
